# Cross-Cultural Comparison of How Mind-Body Practice Affects Emotional Intelligence, Cognitive Well-Being, and Mental Well-Being

**DOI:** 10.3389/fpsyg.2021.588597

**Published:** 2021-07-15

**Authors:** Ofra Walter, Vered Shenaar-Golan, Surekha Routray

**Affiliations:** ^1^Education Department and Social Work Department, Tel Hai Academic College, Qiryat Shemona, Israel; ^2^Social Work Department Tel Hai Academic College, Qiryat Shemona, Israel; ^3^University Khorda Orissa, Bhubaneswar, India; ^4^Head- Social Impact & CSR KIIT-TBI, KIIT University, Bhubaneswar, India

**Keywords:** mental well-being, cognitive well-being, emotional intelligence, culture differences, mind-body practice

## Abstract

The study tested cross-cultural differences between Israeli and Indian participants in the outcomes of mind-body practice (MBP) on emotional intelligence (EI), cognitive well-being, and mental well-being, as well as the predictive role of gender and MBP on cognitive and mental well-being. It drew on a sample of 699 Indian and Israeli participants (ages 18–65) from urban settings and used questionnaires to measure cognitive well-being, demographics, EI, and mental well-being. EI was assessed using the Self Report Emotional Intelligence (SREIT); cognitive well-being was assessed using the Personal Well-being Index; mental well-being was assessed using the Warwick-Edinburgh Mental Well-being Scale (WEMWBS). The effect of demographic variables was tested using the two-sample *T*-test or the Chi-square test. The associations between EI, mental, and cognitive subscales were evaluated using the Pearson correlation coefficient and linear regression with stepwise selection. Results indicated MBP affected EI in both cultures, but Indian participants showed higher EI, and Israeli participants showed lower EI. Israelis also reported higher mental well-being. Future research should examine EI as ability (we examined it as a trait) thus contributing to a better understanding of the similarities and differences between trait and ability EI in different cultures. Future work should also probe gender differences.

## Introduction

Emotional intelligence (EI) is commonly conceptualized as a constellation of emotional perceptions (i.e., trait emotional intelligence) or a set of skills to process emotionally related information (Mayer and Salovey, 1997; Mayer et al., 2001). More precisely, it represents the ability of individuals to make a connection between emotions and reasoning in a way that enables them to use emotions to guide their actions and use reasoning to regulate their emotions (Mayer et al., 2001). People with higher EI cope better with the stressors and hassles of everyday living (Zeidner et al., 2012) and show greater resilience to changes under stress (Schneider et al., 2013). Emotionally intelligent individuals can cope with multiple work demands, readily shift priorities, adapt their responses and tactics to fit fluid circumstances, and respond to a vast variety of emotional stimuli elicited from the inner self and the immediate environment (Sharma, 2011). Those with a high level of EI can achieve a balance between pleasant distractions from aversive events and coming to terms with their moods. Therefore, they can better monitor, reflect and control their emotions, which may contribute to well-being (Salovey et al., 2000).

Emotional intelligence is known to be a good predictor of well-being (Davidson et al., 2000; Sánchez-Álvarez et al., 2016). Well-being has traditionally been measured subjectively by its cognitive and affective components (presence of positive affect and absence of negative affect). The affective and cognitive components are separate in theory but related in actuality (Lucas et al., 1996; Schimmack, 2008). In our study, we looked at cognitive well-being, one aspect of subjective well-being (SWB), by which we mean satisfaction with life based on personal evaluations of one's life in terms of positive versus negative emotions (Lucas et al., 1996), using the Subjective Well-Being Index (SWB-A; International Wellbeing Group, 2006). A more contemporary framework to study well-being is the Warwick-Edinburgh Mental Well-Being Scale (WEMWBS; Tennant et al., 2007), developed to enable the monitoring of mental well-being in the general population and to evaluate projects, programs, and policies which aim to improve mental wellbeing (https://warwick.ac.uk/wemwbs). In our study, we operationalized well-being using both measures, the first for subjective well-being and the second for mental well-being.

Research has found cultural differences in the perception, expression, and regulation of emotions (Eid and Diener, 2001; Nezlek et al., 2008; Koydemir et al., 2013). Emotional experiences and behaviors that fit with a particular culture are likely to be reinforced by that culture. Human societies are typically either individualist, endorsing values such as emotional expression and the right to free choice, or collectivist, placing less emphasis on emotional expression as essential to well-being (Triandis, 1995).

Previous studies have found differences in EI and well-being between individualist and collectivist societies (Furnham and Cheng, 1999; Fukuda et al., 2011, 2012; Li et al., 2012; Martskvishvili et al., 2013). A recent comparative study reported less subjective well-being and lower emotional intelligence for Indian than German students, with the former considered collectivist and the latter individualist (Koydemir et al., 2013). We build on these findings by comparing Israel, an individualist society (Ya' ar and Shavit, 2003) with India, a collectivist society (Suh et al., 1998; Mishra et al., 2006). As each culture is likely to value emotional expression differently, there may be a difference in levels of emotional intelligence, with implications for well-being.

A main difference in our study is that we approached the comparison of EI and well-being in Indians and Israelis through the lens of mind-body practice (MBP). Meditation is a body-mind practice used for emotion regulation, more commonly used in Eastern than Western cultures, but still likely to be practiced in Israel (Patel et al., 2018; Snaith et al., 2018). Most of the research on body-mind practices in Israel and Western countries has focused on stress and trauma release (Ritsner et al., 2000; Franco Justo et al., 2010). Meanwhile, Indian culture is known for its use of body-mind practices, for example, Yoga. Yoga, which is based on meditation, improves mindfulness, self-compassion, and emotion regulation (Walter and Routray, 2020).

We tested the cross-cultural differences between Israel and India and outcomes of MBP on emotional intelligence, cognitive well-being, mental well-being, and the predictive role of gender and MBP on cognitive and mental well-being outcomes in these cultures. We expected Israeli and Indian participants would evidence markedly different levels of EI and well-being (including both cognitive and mental well-being scores), mediated by their use of mind-body practices.

## Methodology

### Participants

The data were collected using the online Qualtrics questionnaire hosted by Survey Monkey. To obtain respondents who regularly practiced MBP, announcements of the study were posted on several major University web sites in India and Israel. Visitors to the websites were informed of the objectives and contributions of the study and given the link to enter the online survey. Participation was voluntary with no monetary compensation. Approximately 6 months after the announcement, a total of 699 questionnaires were completed.

Table 1 presents the demographic description of the sample by country. There were 346 Indian participants, ranging in age from 18 to 65 years old (*M* = 22.0, *SD* = 5.1). Most of the sample, 95%, was <28 years old; 55% were female; 96% were not married, and 92% did not have an academic degree (they were undergraduates at the sampled university). A very small majority, 51%, engaged in mind-body practice. The sample included 353 Israeli participants, ranging in age from 18 to 66 (*M* = 34.7, *SD* = 11.5). However, the rest of the demographic information was quite different as the *P*-value was <0.001 statistically highly significant: 79% of the Israelis were female, 49% were not married, 39% did not have an academic degree, and only 36% engaged in mind-body practice. Table 1 shows the demographic statistics.

**Table 1:** Demographic descriptive statistics by country.

**Variable**	**India *N* = 346**	**Israel *N* = 353**	***P*-value**
Age	22.0 (5.1)	34.7 (11.5)	<0.001
Gender:			<0.001
Female	183 (55.0%)	275 (79.3%)	
Male	150 (45.0%)	72 (20.7%)	
Marital:			<0.001
Married	15 (4.34%)	181 (51.3%)	
Single	331 (95.7%)	172 (48.7%)	
Education:			<0.001
Academic	19 (7.92%)	213 (60.9%)	
Non-academic	221 (92.1%)	137 (39.1%)	
MBP:			<0.001
No	168 (48.8%)	225 (63.9%)	
Yes	176 (51.2%)	127 (36.1%)	

### Measurements

The study used the well-established scales described below. To ensure the validity of the constructs, all questionnaire items originally in English were translated into Hebrew and then back-translated to English by a professional bilingual translator. The back-translations were compared with the original contents to ensure they conveyed the same meaning.

*Emotional intelligence questionnaire*: EI was assessed using the SREIT—Self Report Emotional Intelligence Test (Schutte et al., 1998). SREIT is a 33-item questionnaire that assesses various aspects of EI, based on the three-tier EI model by Mayer and Salovey (1997). The questionnaire uses a five-point Likert scale (1 = strongly disagree; 5 = strongly agree) and assesses three broad dimensions of EI: (1) the appraisal and expression of emotions, 13 items; (2) the regulation of emotions, 10 items; (3) the utilization of emotions, 10 items. SREIT has beneficial predictive and discriminant validity and high reliability, with a Cronbach's alpha (α) of 0.90.

The values of the correlation coefficients between each of the EI subscales for all samples were medium-high, ranging from 0.66 to 0.82. Correlation coefficients for the total score and each one of the subscale scores were high and ranged from 0.88 to 0.91. Because of the high correlation coefficients between the EI subscales and the total score, we decided to use the EI total score as representative of EI in subsequent analysis for all samples. Internal consistency ranged from 0.78 to 0.92.

For Israel, sample values of the correlation coefficients between each of the EI subscales were medium-high and ranged from 0.55 to 0.77. Correlation coefficients between the total score and each one of the subscale scores were high and ranged from 0.82 to 0.91. Internal consistency ranged from 0.71 to 0.90.

For India, sample values of the correlation coefficients between each of the EI subscales were high and ranged from 0.74 to 0.85. Correlation coefficients between the total score and each one of the subscale scores were very high and ranged from 0.92 to 0.94. Internal consistency ranged from 0.82 to 0.94.

*Cognitive well-being*. Cognitive well-being was assessed using the Subjective Well-Being Index (SWB-A; International Wellbeing Group, 2006). The SWB-A is composed of one question inquiring about satisfaction with life as a whole, and eight items measuring satisfaction in specific life domains: standard of living, personal health, achieving in life, personal relationships, personal safety, community-connectedness, future security, and religion. All items were rated on a scale ranging from 0 = completely dissatisfied to 10 = completely satisfied. The internal consistency of the scale was 0.91 for all samples, for the Israeli sample 0.92, and the Indian sample 0.90.

*Mental well-being*. The Warwick-Edinburgh Mental Well-Being Scale (WEMWBS) (Brown, 2020) comprehensively measures mental well-being, including affective-emotional components, cognitive-evaluative components, and items relating to psychological functioning (Tennant et al., 2007). The WEMWBS uses 14 items, such as “I've been feeling confident,” “I've been feeling optimistic about the future,” “I've been feeling relaxed,” “I've been feeling interested in other people.”

Measured on a 5-point Likert type scale ranging from 1 = none of the time to 5 = all of the time. Scores range from 14 to 70, with higher scores indicating higher levels of mental well-being. Criterion validity has been demonstrated through high correlations with well-being measures, such as the PANAS—PA (*r* = 0.71) and the Scale of Psychological Well-Being (*r* = 0.74) (Tennant et al., 2007). Internal consistency based on Cronbach's alpha is 0.91 for this measure (Stewart-Brown et al., 2009) and test-retest reliability after 1 week is 0.83 (Tennant et al., 2007). In this study, Cronbach's alpha for the WEMWBS was 0.92. The internal consistency was 0.96 for all samples, and 0.93 for both the Israeli and the Indian sample.

### Research Procedures

The study used non-probability sampling methods to recruit participants. Prospective participants were approached by the researchers and invited to participate voluntarily. Consenting participants responded to the questionnaire online through the Qualtrics® online survey system, to ensure anonymity and easy access to the questionnaire. The research was conducted under the ethical standards of the [institution-Israel, India] research committees. In India, the study was conducted in English, the university's official language. In Israel, the questionnaires were administered in Hebrew, after they were translated-back translated as described above.

### Statistical Analysis

Continuous variables were reported by mean and standard deviation, and categorical variables were reported by frequency and proportion. Demographic information was compared for Israel and India using the two-sample *T*-test or the Chi-square test. The association between each of the EI, mental, and cognitive subscales was evaluated using the Pearson correlation coefficient. The internal consistency of each scale was achieved by calculating Cronbach's alpha. Because of the high correlation coefficients between the EI subscales and total EI score for both samples, we decided to use the total EI score in all further analysis.

A three-way 2 (MBP: yes vs. no) ^*^2 (country: Israel vs. India) ^*^2 (gender: females vs. males) analysis of variance adjusted to age was run to examine the effect of MBP, country, and gender on the totals for the EI, mental, and cognitive scales. Note that these totals are referred to here as outcome measures. Simple mean analysis was used to reveal significance when an interaction was established.

To find the best subset of demographic parameters that significantly characterized high levels of the outcome measures in each country, we used linear regression with the stepwise selection model method; a 15% Wald chi-square significance level was required for a variable to enter into the model, and a 5% significance level allowed a variable to stay in the model. The candidate variables for the final Indian model were pairwise interactions of MBP with gender and age (most respondents were single and not academic; therefore, we didn't include marital or academic status in the model). The candidate variables for the final Israeli model were pairwise interactions of MBP with gender, age, marital status, and academic status. A *p*-value of 0.05 was considered significant. Statistical analysis was performed by SAS for Windows version 9.4.

## Results

Pearson correlations among the study's measure are presented first for the whole sample and then separately for each country sample. Tables 2A–**C** give the totals of all descriptive statistics and the Pearson correlation coefficients for the whole sample, the Israeli sample, and the Indian sample, respectively. For the whole sample (Table 2A), there was a low positive correlation between the WEMWBS (i.e., mental well-being) and the SWB scale (i.e., cognitive well-being), *r* = 0.25, and also between the WEMWBS and the EI scale, *r* = 0.23. There was a medium negative correlation between the SWB and the EI scale, *r* = −0.43.

**Table 2A:** Totals for scales' descriptive statistics and Pearson correlation coefficients' statistics, *N* = 673.

**Variable**	***N***	***M***	***SD***	**1**	**2**	**3**
1. Total_WEMWBS	599.00	39.51	14.48	(0.96)		
2. Total_SWB	596.00	72.52	21.65	0.25^*^	(0.91)	
3. Total_EI	533.00	2.11	0.55	0.23^*^	−0.43^*^	(0.92)

* *p < 0.001*.

*Cronbach's alpha is in parentheses.

The Israeli sample (Table 2B) showed a medium positive correlation between the WEMWBS and the SWB scale, *r* = 0.64, and a negative correlation between the WEMWBS and the EI scale, *r* = −0.45. There was a medium negative correlation between the SWB and EI scales, *r* = −0.61. The Indian sample (Table 2C) showed a low negative correlation between the WEMWBS and the SWB scale, *r* = −0.16, and a high positive correlation between the WEMWBS and the EI scale, *r* = 0.74. There was a medium negative correlation between the SWB and EI scales, *r* = −0.31 (*N* was change based on particapnt respnds to the questionnaire).

**Table 2B:** Totals for scales' descriptive statistics and Pearson correlation coefficients' statistics for Israeli sample.

**Variable**	***N***	***M***	***SD***	**1**	**2**	**3**
1. Total_WEMWBS	349.00	48.37	9.98	(0.93)		
2. Total_SWB	342.00	74.12	20.79	0.64^*^	(0.92)	
3. Total_EI	251.00	2.2	0.49	−0.45^*^	−0.61^*^	(0.90)

* *p < 0.001*.

*Cronbach's alpha is in parentheses.

**Table 2C:** Totals for scales' descriptive statistics and Pearson correlation coefficients' statistics for Indian sample.

**Variable**	***N***	***M***	***SD***	**1**	**2**	**3**
1. Total_WEMWBS	250	27.13	10.04	(0.90)		
2. Total_SWB	254	70.37	22.63	−0.16^*^	(0.90)	
3. Total_EI	282	2.03	0.59	0.74^**^	−0.31^**^	(0.90)

* *p < 0.05*,

** *p < 0.01*.

*Cronbach's alpha is in parentheses.

To examine the effect of MBP, country, and gender on the EI, WEMWBS, and SWB total scales, we employed a three-way analysis of variance adjusted to age. The F values and the partial Eta^2^ of the three analyses of variance models are presented in Table 3. There was a statistically significant main effect of country on the WEMWBS and the EI scale indicating higher adjusted means for Israel on all scales (WEMWBS: *M* = 47.8 vs. *M* = 28.3, EI: *M* = 2.2 vs. 2.0, *p* < 0.01). There was a statistically significant main effect of gender on the SWB scale, whereby females had higher SWB values than males (*M* = 74.5 vs. *M* = 69.7, *p* < 0.05).

**Table 3:** Analysis of variance results for outcome measures.

**Source**	**^**1**^WEMWBS**	**SWB^**2**^**	**EI^**3**^**
Country	284.66^***^ (0.287)	0.23 (0.000)	14.96^***^ (0.02)
MBP	2.43 (0.003)	0.83 (0.001)	0.01 (0.000)
Country*MBP	0.93 (0.001)	1.62 (0.002)	7.51^**^ (0.010)
Gender	0.15 (0.000)	5.29^*^ (0.007)	0.00 (000)
Country*Gender	0.05 (0.001)	2.95 (0.004)	3.80^*^ (0.005)
MBP*Gender	0.81 (0.001)	0.16 (0.000)	0.23 (0.000)
MBP*Country*Gender	0.22 (0.000)	0.37 (0.000)	0.68 (0.000)

* *p < 0.05*,

** *p < 0.01*,

*** *p < 0.001*.

*One degree of freedom for all factors (1,555)*.

Two degrees of freedom for country, MBP, and country*MBP (1,550).

Three degrees of freedom for country, MBP, and country*MBP (1,485).

There was a statistically significant two-way interaction between MBP and country on EI. Simple main effect analysis revealed that while in Israel, EI adjusted means were not significantly different for those who engaged in MBP and those who did not, in India, respondents engaging in MBP had higher EI values [*M* = 2.1 vs. *M* = 1.9, *t*_(485)_ = −2.45, *p* < 0.05]. There was no significant statistical difference between Indian and Israelis respondents who engaged in MBP, but among those who did not, we found Israelis had higher EI values [*M* = 2.3 vs. *M* = 1.9, *t*_(485)_ = −5.60, *p* < 0.001].

A two-way interaction of country and gender had a statistically significant effect on EI. Simple main effect analysis revealed Israeli males had significantly higher EI values than Indian males [*M* = 2.3 vs. *M* = 1.9, *t*_(485)_ = −3.57, *p* < 0.001], but females did not differ between countries.

Finally, we performed linear regression on the explanatory variables collected in both countries. Tables 4, 5 present the linear regression coefficients of the variables predicting significantly high levels of the three scales (for cognitive well-being, mental well-being, and emotional intelligence) in India and Israel, respectively, as found in the stepwise selection process.

**Table 4:** Linear regression results in India.

	**Dependent variable**		
	**WEMWBS**	**SWB**	**EI**
	**(1)**	**(2)**	**(4)**
Female		8.200^***^	
		(2.862)	
MBP			0.175^**^
			(0.070)
Constant	27.132^***^	65.740^***^	1.952^***^
	(0.635)	(2.117)	(0.048)
Observations	250	243	278

^*^p < 0.05,

** *p < 0.01*,

*** *p < 0.001*.

**Table 5:** Linear regression results in Israel.

	**Dependent variable**		
	**WEMWBS**	**SWB**	**EI**
	**(1)**	**(2)**	**(3)**
Married	3.016^**^	9.133^***^	−0.185^***^
	(1.195)	(2.471)	(0.061)
Academic	3.071^**^	5.635^**^	
	(1.226)	(2.548)	
BMT			−0.170^***^
			(0.064)
Constant	44.974^***^	65.955^***^	2.351^***^
	(0.873)	(1.839)	(0.047)
Observations	345	335	246

^*^p < 0.05,

** *p < 0.01*,

*** *p < 0.001*.

In India, females were related to higher values on the SWB scale (i.e., cognitive well-being) than males (*B* = 8.2, *p* < 0.001) and those engaging in MBP showed higher EI values (*B* = 0.18, *p* < 0.01). In Israel, married individuals (*B* = 3.02, *p* < 0.01) and educated individuals (*B* = 3.07, *p* < 0.01) had higher values on the WEMWBS (i.e., mental well-being). Married (*B* = 9.13, *p* < 0.001) and educated (*B* = 5.63, *p* < 0.01) persons also had higher values on the SWB scale. Married persons (*B* = −0.19, *p* < 0.001) and those engaging in MBP were related to lower values of EI.

## Discussion

We tested the cross-cultural differences between Israeli and Indian participants in the effects of mind-body practice (MBP) on emotional intelligence (EI), and cognitive and mental well-being. We also assessed the predictive role of gender and MBP on cognitive and mental well-being.

We found Israeli participants reported higher EI and mental well-being, but they showed no differences in cognitive well-being. The finding of higher EI in Israeli than Indian participants aligns with findings of previous studies reporting differences in EI between collectivist and individualist cultures (e.g., Koydemir et al., 2013). Furthermore, research on the adaptive use of emotions (Matsumoto et al., 2008) and the efficient use of emotion regulation (Eid and Diener, 2001; Nezlek et al., 2008; Nozaki, 2018) in the context of cultural differences has indicated that individuals from individualistic cultures perceive, express, and regulate emotions more effectively (Karim and Weisz, 2010). These cultural differences are believed to reflect traditional Eastern values which encourage emotional restraint while Western values encourage free and open emotional expression and self-assertion through emotions (Kitayama et al., 2000; Soto et al., 2011). In Western or individualist cultures, such as Israel, high arousal emotions may be more valued and promoted than low arousal emotions. In contrast, in Eastern or collectivist cultures, such as India, low arousal emotions may be more valued (Lim, 2016).

The study looked at both cognitive and mental components of well-being. It found higher levels of mental well-being but not cognitive well-being among Israeli participants. The opposite was true for the Indian sample. Previous studies have found that individualist societies tend to have higher levels of overall well-being; one explanation is that cultures differ in the value they place on personal happiness (Diener et al., 1995). As a collectivist culture, India emphasizes values of group cohesion and places less emphasis on the role of inner disposition in self-conception or the need for individual happiness (Mishra et al., 2006; Taggart et al., 2013). In contrast, by endorsing individualistic values, the Israeli culture values the freedom to pursue personal fulfillment, and this, in turn, may have a positive impact on mental well-being (Suh et al., 1998; Veenhoven, 1999). Our study corroborates this suggestion. Other studies have also found that people in individualistic countries report more positive affect than their counterparts in collectivistic countries (Diener et al., 1995; Kitayama and Markus, 2000). For instance, Gökçen et al. (2014) found British participants (i.e., individualistic) scored higher than Chinese or Japanese participants (collectivist) on constructs associated with trait EI, such as happiness and psychological well-being.

The finding of the absence of differences in the level of the cognitive component of well-being suggests a cultural element common to both countries: a strong familial culture. Israel is often called a “child-oriented” society (Scharf, 2014), and India values a strong emotional dependence on family (Mishra et al., 2006).

For both cultures, we found a negative correlation between the cognitive component of SWB and EI. The correlation between mental well-being and EI was different, with a negative correlation for the Israeli sample and a high positive correlation for the Indian sample. These results substantiate the current understanding of cultural similarities and differences in EI (Peña-Sarrionandia et al., 2015; Mestre et al., 2016). Previous studies on cultural differences in emotional intelligence have revealed inconsistencies. Whereas some studies (e.g., Gallagher and Vella-Brodrick, 2008) found EI was more closely related to the affect component than to life satisfaction, others (e.g., Zeidner and Olnick-Shemesh, 2010) failed to find such an association. From a cultural perspective, the relations between emotional intelligence and well-being may reflect the different patterns and different mechanisms operating in various cultures.

According to Koydemir et al. (2013), EI is relevant for accurately perceiving, expressing, and regulating emotions and effectively coping with problems; thus, higher EI is likely to result in more well-being. Although the EI of Israeli participants was high, their cognitive and mental well-being was not. It may that stressful daily life does not allow them to achieve a balance in their positive and negative emotions (Koydemir et al., 2013). Arguably, they might pay close attention to their emotions but lack the ability to repair their negative moods in a day-to-day context. This possibility is supported by previous studies connecting emotional intelligence to life satisfaction, defined as the cognitive component of subjective well-being (e.g., Martinez-Pons, 1997; Extremera and Ferna'ndez-Berrocal, 2005; Gallagher and Vella-Brodrick, 2008).

In our study, the Israeli results showed a positive correlation between mental and cognitive well-being; the opposite was true for the Indian results. This suggests different components comprise well-being in the different cultures with different effects on cognitive well-being and EI. Overall, the Israeli participants demonstrated a higher EI; this was expected, as it reflects Western societies' greater engagement in emotional expression.

The regression analysis supported the role of MBP in EI. There was no significant statistical difference between Indian and Israelis participants who engaged in MBP. However, the Indian participants who practiced MBP demonstrated a higher EI than the Indian participants who did not. Meanwhile, in Israel, engaging in MBP was related to lower EI. In Israel, MBP is not part of the culture but has been adopted by some as a practice to cope with life stressors, as it offers a strategy to connect to the inner self. It seems the inner connections deepen emotional understanding, but there might be an inability to change negative emotions to positive ones. In India, MBP is part of the experience of life, and, as such, may affect the emotional intelligence of those who practice MBP regularly, enabling them to connect to the inner self and understand their emotions. Our findings suggest the differences of EI mechanisms used in different cultures, including the ability of MBP to increase well-being.

Results for gender showed Israeli males had higher significant EI values than Indian males, and this pattern of results mirrored the overall difference for the cultures. Indian females had higher values on the SWB scale (i.e., cognitive well-being) than Indian males (De Vibe et al., 2013). Note that the regression analysis of the Israel participants revealed some findings not essential to this study; they will be discussed in future work.

To conclude, researchers have repeatedly emphasized the importance of determining whether the EI construct could be generalized to diverse groups in Western and Eastern cultures (e.g., Gohm, 2004; Ekermans, 2009), and previous studies have found that trait EI measures apply to both Western and Eastern populations (Koydemir and Schütz, 2012; Gökçen et al., 2014; Nozaki and Koyasu, 2016). However, it remains unclear whether the psychological processes underlying the EI construct differ for Western and Eastern populations. While previous studies show trait EI measures apply to both Western and Eastern populations (Koydemir and Schütz, 2012; Gökçen et al., 2014; Nozaki and Koyasu, 2016), our findings suggest other cultural components should be considered. Furthermore, it seems MBP makes different contributions to EI depending on the culture.

## Conclusion

We tested the cross-cultural differences between Israeli and Indian participants in the effects of mind-body practice (MBP) on emotional intelligence (EI), and cognitive and mental well-being. Our assumption was that Israel is an individualist society, and India is a collectivist one, and we wanted to see if this made a difference in the results. We also assessed the predictive role of gender and MBP on cognitive and mental well-being.

The two participating cultural groups showed no differences in the cognitive component of well-being, but Israeli participants reported higher EI and mental well-being. Arguably, traditional Eastern values (India) encourage emotional restraint while Western values (Israel) encourage emotional expression. Moreover, for the Israeli participants, EI and mental well-being were negatively correlated, but we found the opposite results for the Indian participants. Israelis may be able to pay close attention to their emotions but lack the ability to cope with real stress.

MBP affected EI in the same way in both cultures. However, Indian participants who engaged in MBP had higher EI than Indians who did not comparators, but Israeli participants who engaged in MBP had lower EI than Israelis who did not. It is possible that Israelis use MBP to cope with life stressors, but for Indians, MBP is part of the culture.

Interestingly, gender differences were not salient, with only one finding of difference: Indian females indicated higher cognitive-well-being than Indian males.

### Limitations and Future Research

Admittedly, the research had some limitations. Future work should focus on recruiting matching samples, including such demographic variables as education status, a limitation of this study. While provocative, our findings require replication and further investigation across other cultures. Future work may also consider using more cultural domains at the country level as supplements to an individual-level cultural self-conception measure (e.g., Nezlek et al., 2008). Other research could examine EI as an ability, thus contributing to a better understanding of the similarities and differences between trait and ability EI in different cultures. Finally, it would be useful to expand the understanding of gender differences.

## Data Availability Statement

The raw data supporting the conclusions of this article will be made available by the authors, without undue reservation.

## Ethics Statement

The studies involving human participants were reviewed and approved by Tel Hai Academic College ethical committee. The patients/participants provided their written informed consent to participate in this study.

## Author Contributions

All authors listed have made a substantial, direct and intellectual contribution to the work, and approved it for publication.

## Conflict of Interest

The authors declare that the research was conducted in the absence of any commercial or financial relationships that could be construed as a potential conflict of interest.
